# 

**Published:** 1923-03

**Authors:** 


MARCH, 1923.

*Vol. XL.* *No.* 148.

### THE BRISTOL MEDICO-CHIRURGICAL JOURNAL

PUBLISHED UNDER THE AUSPICES OF

*THE BRISTOL MEDICO-CHIRURGICAL SOCIETY.*

*Editor:* P. WATSON-WILLIAMS, M.D.,

WITH WHOM ARE ASSOCIATED

JAMES SWAIN, C.B., C.B.E., M.S., M.D.,

J. LACY FIRTH, M.S., M.D., CAREY COOMBS, M.D.

J. A. NIXON, C.M.G., M.D., *Assistant Editor.*

*Editorial Secretary:* A. L. FLEMMING, M.B., B.Ch.

BRISTOL: J. W. ARROWSMITH LTD.

LONDON: SIMPKIN, MARSHALL, HAMILTON, KENT & CO. LTD.

*Price Three Shillings net.*

